# Supplementary material for: Hypothetical acceptability of hospital-based post-mortem pediatric minimally invasive tissue sampling in Malawi: The role of complex social relationships
Source: PLoS One. 2021 Feb 4;16(2):e0246369. doi: 10.1371/journal.pone.0246369 (PMC7861399; doi:10.1371/journal.pone.0246369)
Supplement: S1 Appendix — (DOC) [file pone.0246369.s001.doc]

**MITS in Malawi**

**Discussion Guide: Frontline Clinical Staff**

Determining acceptability and improving cultural appropriateness of approach

**Target participants for series of group discussions: Health Care Workers, pathologist(s), technicians or assistants, frontline nursing staff, referring paediatricians, administrators, community health workers, palliative care staff, translators, senior ward managers**

1. Welcome and introductions

Welcome and thank you for taking time to discuss this topic. The purpose of our conversation today is to learn more about your experiences surrounding determining the cause(s) of death in a child who has died in hospital. As you know, sometimes the cause of death is unclear to clinicians – there may be multiple factors involved, the child may not have been diagnosed prior to death, and even if diagnosed, there can be limitations to our diagnostic tests. Post-mortem examinations, such as autopsy, can help determine cause of death in such circumstances. However, there are sometimes personal, psychological and cultural barriers to performing full autopsy, especially in a child who has died. A technique which is gaining in use as an alternative to full autopsy is ‘minimally invasive tissue sampling’. This involves a technique similar to a needle biopsy, where tissue samples are taken from critical organs for laboratory examination to determine cause of death. Organs sampled might include lung, heart, liver, kidney, or brain. For the one that will be conducted at QECH, there will also be an intestinal sampling using endoscopy.

1. Just so that we have a sense of everyone’s experience before we begin, how many of you have heard of MITS, or minimally invasive tissue sampling?

Probe: Direct experience of autopsy

Probe: Experience of MITS

1. Are there any special concerns that you as a health care worker would have related to the use of MITS in determining cause of death in children?
2. What issues or concerns might parents have about MITS procedure following the death of a child?

Probe: endoscopy

Probe: presence of a community/family member in performing MITS

1. What are some of the concerns you might have in approaching parents for post-mortem examination using MITS?
2. How would you address these concerns in your approach to parents who have just lost a child? In your experience what are some sensitive ways to approach parents?

Probe: communication

Probe: supportive actions (e.g., sitting with parents, consoling parents, visiting home to give condolences, referral to grief support, paying for funeral, other?)

Probe: Peer support

Probe: Mother vs. Father vs. Grandparent or other – how to involve different family members

Probe: Timing: can it even be done at night, how long after death?

1. What are some concerns that members out in the community might have related to the use of MITS in determining cause of death in children?

Probe: Variation across different communities or cultural/religious groups?

Probe: Specific examples of beliefs or taboos?

Probe: What happens when rumours spread? How should this be managed and who should be responsible for addressing community concerns or rumours?

1. Do you have suggestions or ideas for addressing possible community concerns about MITS?
2. Do you have any suggestions for staff training and sensitization around MITS?
3. Do you have any other thoughts you wish to share on this topic?
