# Supplementary material for: Hypothetical acceptability of hospital-based post-mortem pediatric minimally invasive tissue sampling in Malawi: The role of complex social relationships
Source: PLoS One. 2021 Feb 4;16(2):e0246369. doi: 10.1371/journal.pone.0246369 (PMC7861399; doi:10.1371/journal.pone.0246369)
Supplement: S2 Appendix — (DOC) [file pone.0246369.s002.doc]

**MITS in Malawi**

**Discussion Guide: Hospital support staff**

Determining acceptability and improving cultural appropriateness of approach

**Target participants for series of group discussions: Guards, cleaners, patient attendants**

1. Welcome and introductions

Welcome and thank you for taking time to discuss this topic. The purpose of our conversation today is to appreciate your understanding of a relatively new process in establishing the cause of death among children which is called minimally invasive tissue sampling (MITS). We would like to understand how acceptable the procedure would be among community members and how best can parents who have just lost a child be approached for the procedure.

1. What are some of the ways you have heard about that we can find out what has caused a child’s death?
2. What issues or concerns might parents have about MITS procedure following the death of a child?

Probe: endoscopy

Probe: presence of a community/family member in performing MITS

1. Who would be the right person to approach for MITS consent in a family in the event of a death?
2. How do you think parents would want to be approached if they had just lost a child?

Probe: communication

Probe: supportive actions (e.g., sitting with parents, consoling parents, visiting home to give condolences, referral to grief support, paying for funeral, other?)

Probe: Peer support

Probe: Mother vs. Father vs. Grandparent or other – how to involve different family members

Probe: Timing: is night appropriate?

Probe: Information they may want to be told

1. What are some concerns that members in the community might have related to the use of MITS in determining cause of death in children?

Probe: Variation across different communities or cultural/religious groups?

Probe: Specific examples of beliefs or taboos?

Probe: What happens when rumours spread? How should this be managed and who should be responsible for addressing community concerns or rumours?

1. Do you have suggestions or ideas for addressing possible community concerns about MITS?
2. Do you have any suggestions for staff training and sensitization around MITS?
3. Do you have any other thoughts you wish to share on this topic?
