# Supplementary material for: Hypothetical acceptability of hospital-based post-mortem pediatric minimally invasive tissue sampling in Malawi: The role of complex social relationships
Source: PLoS One. 2021 Feb 4;16(2):e0246369. doi: 10.1371/journal.pone.0246369 (PMC7861399; doi:10.1371/journal.pone.0246369)
Supplement: S3 Appendix — (DOC) [file pone.0246369.s003.doc]

**MITS in Malawi**

**Discussion Guide: CHAIN team**

Determining acceptability, improving cultural appropriateness of approach and concerns that CHAIN team might have over MITS

**Target participants for group discussions: administrators, clinicians and lab technicians**

1. Welcome and introductions

Welcome and thank you for taking time to discuss this topic. The purpose of our conversation today is for us to discuss on MITS which is a sub-study that is expected to be conducted in the CHAIN study. As you know, sometimes the cause of death is unclear to clinicians – there may be multiple factors involved, the child may not have been diagnosed prior to death, and even if diagnosed, there can be limitations to our diagnostic tests. Post-mortem examinations, such as autopsy, can help determine cause of death in such circumstances. However, there are sometimes personal, psychological and cultural barriers to performing full autopsy, especially in a child who has died. A technique which is gaining in use as an alternative to full autopsy is ‘minimally invasive tissue sampling’.

1. Just so that we have a sense of everyone’s experience before we begin, how many of you have heard of MITS, or minimally invasive tissue sampling?

Probe: Direct experience of autopsy

Probe: Experience of MITS

1. Are there any special concerns that you would have in the use of MITS under CHAIN?

Probe: implications on recruitment into the CHAIN study

Probe: Rumours

1. How would such concerns be addressed?
2. What issues or concerns might parents have about MITS procedure following the death of a child?

Probe: endoscopy

Probe: presence of a community/family member in performing MITS

1. What are some concerns that members out in the community might have related to the use of MITS in determining cause of death in children?

Probe: Variation across different communities or cultural/religious groups?

Probe: Specific examples of beliefs or taboos?

Probe: What happens when rumours spread? How should this be managed and who should be responsible for addressing community concerns or rumours?

1. Do you have suggestions or ideas for addressing possible community concerns about MITS?
2. Do you have any suggestions for staff training and sensitization around MITS?
3. Do you have any other thoughts you wish to share on this topic?
