# Supplementary material for: Hypothetical acceptability of hospital-based post-mortem pediatric minimally invasive tissue sampling in Malawi: The role of complex social relationships
Source: PLoS One. 2021 Feb 4;16(2):e0246369. doi: 10.1371/journal.pone.0246369 (PMC7861399; doi:10.1371/journal.pone.0246369)
Supplement: S5 Appendix — (DOC) [file pone.0246369.s005.doc]

**MITS in Malawi**

**Discussion Guide: Religious leaders**

Determining acceptability and improving cultural appropriateness of approach

**Target participants for this discussion: religious leaders**

1. Welcome and introductions

Welcome and thank you for taking time to discuss this topic. The purpose of our conversation today is to appreciate your understanding of bereavement traditions in your various religions. We would like to understand, also, how your religion would be accommodating to a relatively new process in establishing the cause of death among children which is called minimally invasive tissue sampling (MITS). We will get to understand the process through the discussion as we will also appreciating its acceptability among your congregation basing on your respective religious doctrine.

1. If you or someone in your community had lost a child, would you or those in your community want to understand what has caused the death of the child?

Probe: Circumstances that would make a parent want to know cause of death?

1. What are some of the ways you have heard about that we can find out what has caused a child’s death?
2. If there was a way of establishing the cause of death through the sampling of body tissues (describe sampling as it is done on sick people), would your religion be acceptable to it?

Probe: Reasons

1. Are there any special concerns that your religion would have related to the use of MITS in determining cause of death in children?

Probe: concerns on specific body parts i.e. endoscopy

1. What are some concerns that members out in the community/your congregation might have related to the use of MITS in determining cause of death in children?

Probe: Variation across different communities or cultural/religious groups?

Probe: Specific examples of beliefs or taboos?

Probe: What happens when rumours spread? How should this be managed and who should be responsible for addressing community concerns or rumours?

1. Do you have suggestions or ideas for addressing possible congregation concerns about MITS?
2. What practices around death does your religion have?

Probe: burial practices

Probe: Life after death

Probe: MITS process in relation to burial practices

1. How best can parents who have just lost a child be approached in a hospital setting?
2. Do you have any other thoughts you wish to share on this topic?
