# Supplementary material for: Hypothetical acceptability of hospital-based post-mortem pediatric minimally invasive tissue sampling in Malawi: The role of complex social relationships
Source: PLoS One. 2021 Feb 4;16(2):e0246369. doi: 10.1371/journal.pone.0246369 (PMC7861399; doi:10.1371/journal.pone.0246369)
Supplement: S6 Appendix — (DOC) [file pone.0246369.s006.doc]

# MITS in Malawi

**Discussion Guide: Parents Round 2**

Determining acceptability and improving cultural appropriateness of approach

**Target participants for this discussion: Parents**

1. Welcome and introductions
2. If you had a child who was hospitalised that unfortunately dies while under hospital care, would you want to understand what has caused the death of the child?

Probe: Circumstances that would make a parent want to know cause of death?

Probe: reasons for the interest in understanding CoD (personal vs public health benefits)

Probe: impact on future treatment seeking

Probe: the difficulty of others to understand the CoD (make reference to civic education vs formal education)

Probe: where do people get health information?

1. What are some of the ways you have heard about that we can find out what has caused a child’s death?

Probe: Concerns with the ways

Probe: Sources of information on the ways

1. If there was a way of determining cause of death using a method that is the same as tissue sampling (e.g. sampling blood) how open would you be to it?

Probe: Previous experience with testing body in time of hospitalisation

Probe: Decision making in previous experience

Probe: Information (sources of information) in previous experience

1. Are there specific body parts you would not want sampled?

Probe: Endoscopy, brain

1. Who would be the right person to approach the family for MITS consent in the event of a death: why?

Probe: Trust (the factors influencing that trust)

Probe: Social relationship (healthy and ‘coerced’ relationships)

Probe: Power hierarchies (rights and responsibilities)

Probe: Examples of healthy and unhealthy relationships

Probe: what are the factors that would influence whom you would want to approach you?

Probe: what is considered appropriate treatment in a hospital; how would it impact on MITS acceptability?

1. In your family, who would be the person to be approached for consent?
2. How would you want to be approached if it happened that you are a parent/guardian/relation who has just lost a child?

Probe: supportive actions (e.g., sitting with parents, consoling parents, visiting home to give condolences, referral to grief support, paying for funeral, other?)

Probe: Information they may want to be told

1. What are some concerns that members in the community might have related to the use of MITS in determining cause of death in children?

Probe: Variation across different communities or cultural/religious groups?

Probe: Specific examples of beliefs or taboos?

Probe: What happens when rumours spread? How should this be managed and who should be responsible for addressing community concerns or rumours?

Probe: Influence of cultural/religious concerns on MITS acceptability

Probe: Rural/Urban divide (the fluidity in the movement between the spaces) and impact on acceptability

Probe: Fatalism

1. Do you have any other thoughts you wish to share on this topic?
