# Supplementary material for: Hypothetical acceptability of hospital-based post-mortem pediatric minimally invasive tissue sampling in Malawi: The role of complex social relationships
Source: PLoS One. 2021 Feb 4;16(2):e0246369. doi: 10.1371/journal.pone.0246369 (PMC7861399; doi:10.1371/journal.pone.0246369)
Supplement: Attachment — Submitted filename: Response to Reviewers.docx [file pone.0246369.s008.docx]

January 15, 2021

Ritesh G. Menezes, M.B.B.S, M.D., Diplomate N.B.

PLOS ONE

Re: PONE-D-20-25933

Dear Dr. Menezes:

Thank you for the opportunity to resubmit our manuscript PONE-D-20-25933 with minor revisions. We appreciate the detailed review once again provided by you and the reviewers. We have responded to each comment in an itemized list below. All edits to the manuscript have been marked in track changes. The revised version of the manuscript that we are resubmitting has been reviewed and approved by all co-authors.

Sincerely,

Sarah Lawrence, MPH

| Comment | Response |
| --- | --- |
| REVIEWER 1 | |
| The manuscript addresses a critical issue related to the MITS conduct in Malawi. The formative research findings must be used for shaping the conduct of the MITS and obtaining consent for MITS. The authors should comment on use of the findings in the discussion section. | Thank you for this comment, in the discussion section we note the following on lines 513-516: “…this formative research provided a foundation for implementation of a MITS study to elucidate CoD among hospitalized young Malawian children. Important lessons learned informed development of consent and study procedures.” Details of our specific approaches are outlined in Table 2 (pp 19-20) in the 3^rd^ column. |
| REVIEWER 2 | |
| Well done on revisions. Two minor suggestions: Line 117 is 'expectances' a typo? Unsure of the meaning of that word. | Thank you for this feedback, we have updated the word “expectances” to read “expectations” in line 117. The full text now reads “Furthermore, consent and reported satisfaction reflect power relations and expectations more than acceptance.” |
| Line 443: incomplete or interrupted sentence immediately following Table 3 | Thank you, we have deleted this text from line 443. |
| REVIEWER 3 | |
| I have reviewed the manuscript and all suggestions have been addressed. There is a mistake in line 269: It reads “ethic groups” instead of “ethnic groups”. I am satisfied with the current version of the manuscript. | Thank you, we have updated the text appropriately to read “ethnic groups” in line 269. |
